# Associations between biopsychosocial factors and transportation patterns of older adults residing in Klang Valley, Malaysia

**DOI:** 10.3389/fpubh.2023.1153822

**Published:** 2023-05-10

**Authors:** Nurul Syuhada Mohd Rosnu, Wan Syafira Ishak, Mohd Harimi Abd Rahman, Suzana Shahar, Charles Musselwhite, Arimi Fitri Mat Ludin, Tengku Aizan Hamid, Abdul Rais Abdul Latiff, Devinder Kaur Ajit Singh

**Affiliations:** ^1^Center for Healthy Ageing and Wellness (H-Care), Faculty of Health Sciences, Universiti Kebangsaan Malaysia, Kuala Lumpur, Malaysia; ^2^Optometry and Vision Sciences Programme, Center for Rehabilitation and Special Needs, Faculty of Health Sciences, Universiti Kebangsaan Malaysia, Kuala Lumpur, Malaysia; ^3^Centre for Ageing and Dementia Research, Aberystwyth University, Aberystwyth, United Kingdom; ^4^Malaysian Research Institute on Ageing, Universiti Putra Malaysia, Serdang, Selangor, Malaysia; ^5^School of Social Sciences, Universiti Sains Malaysia, Gelugor, Pulau Pinang, Malaysia

**Keywords:** aging, biopsychosocial factors, transportation patterns, outdoor mobility, older driver

## Abstract

**Introduction:**

Aging is associated with physiological changes in multiple systems in the body and may impact the transportation choices of older adults. In this study, we examine the associations between biopsychosocial factors and the transportation choices of Malaysian older adults.

**Methods:**

One hundred and nineteen (119) older adults, aged 60 and above, living in Klang Valley, Malaysia were recruited for this cross-sectional study. Participants were interviewed face-to-face to obtain sociodemographic data, health status (whether there were and, if yes, the number of comorbidities), outdoor mobility and transportation patterns, Instrumental Activity Daily Living (IADL) status and cognitive function. Participants’ physical performance (dominant handgrip strength, 10-m walk, and timed up and go tests), hearing threshold (pure tone audiometry), and vision function (visual acuity, contrast sensitivity) were measured. Transportation patterns of older adults were categorized into three groups, that is, flexible (using public transport and/or private vehicles), using only private vehicles and restricted (relying on others or walking).

**Results:**

Further information is needed to enable such older adults as older women, those with comorbidities and poorer functional status to access transportation, especially to meet their health care needs.

**Discussion:**

The majority (51%) of participants were in the ‘using only private vehicles’ group, followed by the ‘flexibles’ (25%) and the ‘restricted’ (24%). Factors significantly associated with the restricted transportation group were: (a) being female (AdjOR 15.39, 95% CI 0.86–23.39, *p* < 0.001); (b) being Malay (AdjOR 21.72, 95% CI 0.36–16.12, *p* < 0.001); (c) having higher number of comorbidities (AdjOR 14.01, 95% CI 0.20–13.21, *p* = 0.007); and (d) being dependent in IADL (AdjOR 13.48, 95% CI 0.51–1.78, p = 0.002).

## 1. Introduction

The world’s population of older adults is expected to double from 12 to 22% between 2015 and 2050 (1). Approximately 80% of these older adults will be living in low- and middle-income countries (1). Malaysia’s population is multiethnic – the majority are from the Malay ethnic group (69.6%), followed by the Chinese (22.8%) and the Indians (6.6%). Malaysia’s Elderly National Policy follows the definition of ‘older adults’ adopted by the United Nations World Assembly on Ageing, that is, ‘older adults’ are individuals aged 60 years and above (2). Notably, 7.3% of Malaysia’s population are now older adults and Malaysia is expected to become an aged country by 2050 when 15% of the total populations are among older adults (2).

The Klang Valley, a densely populated area on the west coast of Malaysia, is a particularly appropriate venue for this study. As Klang Valley expands rapidly, diverse choices of private and public transportation such as car service, taxis, busses and light rail have become available. But whether the older adults residing in Klang Valley are able to take advantage of these transportation choices to participate in out-of-home activities, thereby aging healthily and having a good quality of life (QoL) (3), depends on various biopsychosocial factors.

An older individual’s mobility, that is, their ability to move purposively in their environment (4), is impacted by the physiological changes and decline in functional capacity associated with aging (5). Some factors commonly associated with increased outdoor mobility include: gender, level of education, marital status, and access to transportation (6). Morbidities, physical, cognitive, nutritional, hearing and visual issues also impact mobility (7-11). The prevalence of multimorbidity among Malaysian older adults is 40.6% (12, 13). Older individuals with multimorbidity experience restrictions in outdoor mobility and access to transportation, among other issues (14). But when older adults participate in more out-of-home activities, they become more independent. For example, older adults who go outdoors at least four times a week are more likely to remain independent in their activities of daily living (ADL) (15).

As for transportation patterns, older adults were reported to be more dependent on private vehicles, compared to public transportation (16). A study by (17) found that adults who possessed a private vehicle engaged in out-of-home activities two times more a week, compared to those without their own vehicles and the majority of these adults were men. However, for adults older than 70 years, there was a significant negative association between self-driving and age. In Georgetown, Malaysia, the ‘young’ old (60–64 years old) and ‘older’ adults (65–75 years old) were more likely to travel using private vehicles as passengers or drivers, while adults aged 76 and above were more likely to walk or cycle (18). In addition, being female, having a disability that impacted mobility and using an assistive walking device were common factors that negatively impacted an adult’s ability to be independent in their transportation (19).

Moreover, older adults’ need to access transportation for healthcare services increases with age (4, 20). On average, older Malaysian adults reportedly visit a hospital as outpatients for 5.92 visits per year (21). Failure to access healthcare-related services due to transportation and outdoor mobility barriers may result in delayed care and poor management of chronic illnesses, leading to adverse health outcomes (22). Navarrete-Reyes et al. (19) studied the transportation needs of older adults requiring outpatient follow up and found that 46% of the participants reported difficulties accessing transport. Transportation has been identified as a major barrier to accessing healthcare services among older adults in Southeast Asia (23).

Considering the numerous biological, psychological, and social factors which impact the transportation needs of older adults, we aim to determine the associations between these biopsychosocial factors and the transportation choices of older Malaysian adults residing in the Klang Valley. The biopsychosocial model considers the complex interaction between biological, psychological, and social factors in understanding a pattern (24). While this model has been used extensively in medical studies, it has not been used to study the transportation choices made by older adults. Using the biopsychosocial model will help us understand much better older adults’ dependency on transportation from the integration of biological, individual and social perspectives (25).

## 2. Methods

### 2.1. Study design

This cross-sectional descriptive study was part of the Consortium on Mobility and Transportation in an Ageing Society (CoMTAS) project. Older adults aged ≥60 years were recruited from Selangor state and Kuala Lumpur between November 2021 and August 2022. This study was approved by the Research Ethics Committee of Universiti Kebangsaan Malaysia (UKM PPI/111/8/JEP-2021-742) and was conducted in accordance with the Helsinki Declaration. The participants provided written informed consent before the study commenced.

### 2.2. Participants

G*Power version 3.1.9.3 was used to determine the minimum number of participants required to enroll in this study. Under test family, *F* tests was selected, with effect size 0.12 and α error of 0.05 and 85% power. A total of 20% missing data was anticipated. The sample size required for this cross-sectional study was 129. Inclusion criteria included: living in Klang Valley, being aged 60 or above, being able to ambulate with or without assistive devices, and being able to understand and speak Malay, English or Mandarin Chinese. Individuals with documented major psychiatric illness or mental disorder were excluded from the study. Individuals who met the inclusion criteria were invited to participate in the study on a specific date. Participants who met the criteria were invited to participate in the study on an appointed date.

### 2.3. Data collection

Posters and flyers were used to publicize the study at such venues as the Audiology Clinic, Faculty of Health Sciences, Universiti Kebangsaan Malaysia (UKM), Hospital Chancelor Tuanku Mukhriz and several Klang Valley communities. The period of recruitment of participants was from November 2021 to July 2022.

During the data collection session, the participants completed a consent form and were briefed on the objectives and procedures of the study. A trained research team member (NSMR) then used a structured questionnaire to obtain the participants’ socio-demographic data, medical history, outdoor mobility and transportation patterns, the Lawton Instrumental Activities of Daily Living (IADL) status. The participants’ cognitive functions were then screened using the Identification of Dementia in Elderly Africans (IDEA). The Craig Hospital Inventory of Environmental Factors (CHIEF) was used to assess the impact of various environmental factors on the participants. Tympanometry and pure tone audiometry (PTA) tests were administered to determine the participants’ hearing thresholds. Participants were then tested for visual acuity and contrast sensitivity and handgrip strength (HGS). They then performed the timed up and go (TUG) and 10-metre walk (10 mW) tests and completed the Mini-Nutritional Assessment Scale Short Form (MNA-SF), Geriatric Depression Scale (GDS) and SARC-F questionnaire. The questionnaires and clinical tests were administered randomly to avoid sequence bias and lasted for 1.5 h. Each participant was interviewed and assessed by the same research team member (NSMR) to reduce errors.

**Figure 1:**
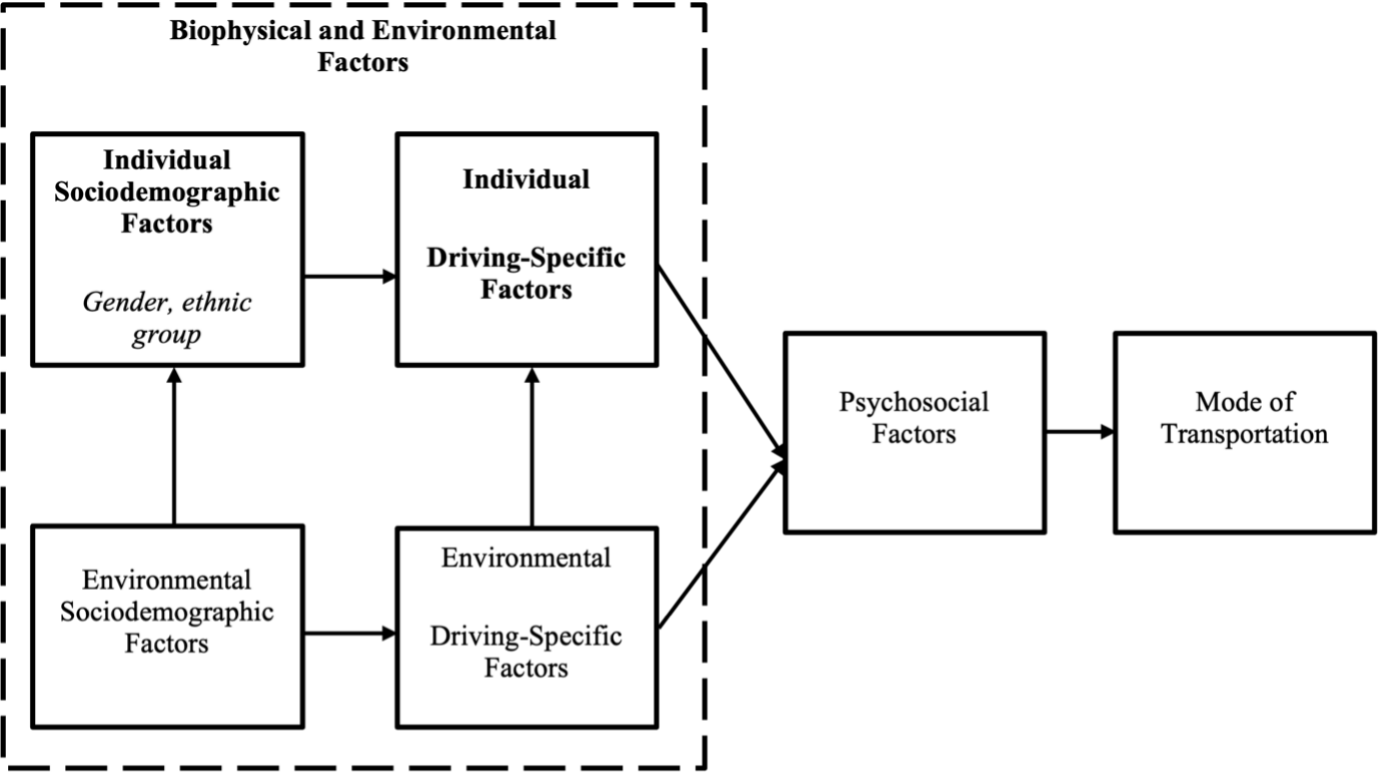
Conceptual framework adapted from the Multilevel Older Persons Transportation and Road Safety (MOTRS) with significant associated factors with transportation patterns in bold.

Figure 1 shows the framework adapted from the Multilevel Older Persons Transportation and Road Safety (MOTRS) (26). In this study, the multilevel MOTRS model is divided into four levels: (a) sociodemographic variables, (b) driving-specific variables, (c) psychosocial variables, and (d) mode of transportation. The sociodemographic and driving-specific variables represent the biophysical and environmental factors from the biopsychosocial model.

#### 2.3.1. Socio-demographic data, medical history

The information gathered included: age, gender, ethnicity, years of education, education level, monthly income, occupation, fear of falling, chronic medical conditions, and self-reported exercise. For self-reported exercise, participants were asked if they engaged in physical activity for 20 min, three times a week.

#### 2.3.2. Nutritional assessment

The MNA-SF was used to assess the nutritional status of the participants. A total score of <8, 8–11, and >11 indicated malnutrition, at risk of malnutrition, and normal nutritional status, respectively, (27). MNA-SF is a valid and reliable tool to screen for malnutrition among older adults with Cronbach’s α coefficient of 0.80 (28).

#### 2.3.3. Audiometric assessment

Pure tone audiometry test was carried out in a sound-proof booth using a calibrated AC 40 Interacoustic clinical audiometer equipped with TGH 39 headphones and E-A-R Tone 3 A insert earphones. The air-conduction thresholds for each ear were obtained monaurally at octave and half octave frequencies from 250 to 8,000 Hz. In this study, we categorized the hearing thresholds in the better ear as pure-tone average for the octave frequencies from 0.5 to 4 kHz and high-frequency pure tone average (HFPTA) for the octave from 2 to 8 kHz. Participants with frequency average of >40 dBHL were categorized as having ‘hearing loss.’ The PTA test is the gold standard for determining type and degree of hearing loss and is considered reliable with test–retest variations within the 5–10 dB range (29).

#### 2.3.4. Cognitive screening assessment

The risk of dementia in participants was measured using the IDEA screening tool. This tool is available in Malay and English and is validated and adjusted according to cultural norms in Malaysia (30). The six items assessed were abstract thinking, spatial orientation, temporal orientation, language fluency, delayed recall and praxis. The IDEA cognitive screen showed moderate internal consistency with a Cronbach’s α coefficient of 0.686 (30).

#### 2.3.5. Visual assessment

Participants’ visual acuity was measured using the Tumbling E folding distance chart at 3 m. The E chart had orientations of the letter E in four different directions: right, left, up and down. The chart was positioned at the participant’s eye level, while measuring visual acuity. During the assessment, the participant stood three meters from the chart and stated the E’s direction, whether it was facing up, down, left or right. Visual acuity in both eyes was taken separately and the better eye was established by choosing the eye with lower LogMAR value. Low vision was defined as having visual acuity in the better eye of more than 0.3 LogMAR. The Pelli Robson contrast sensitivity chart was used to measure habitual monocular and binocular contrast sensitivity at 1 m and recorded in Log Contrast Sensitivity units.

#### 2.3.6. Physical performance assessment

Participants’ dominant HGS, TUG, and 10 mW tests were measured. HGS was evaluated using handgrip dynamometer (Jamar Hydraulic Dynamometer, Wisconsin, United States) with the participant sitting and upper limb positioned, with the elbow flexed at 90 degrees, unsupported. Using the dominant hand, two trials were taken, and the highest score was recorded, in kilograms. It is currently the gold standard tool for measuring grip strength with excellent validity and reliability (31). For the TUG, the participants were asked to rise from a 46 cm highchair, walk forward at their normal pace for three meters, turn 180̊, return to the chair and sit down (32). The mean of TUG sessions was recorded in seconds (s). For this study, if the time taken to complete TUG ≥10.2 s, the participant was considered as having the physiological risk of falling (33). The TUG has demonstrated validity for assessing functional mobility with excellent reliability (34).

As for the 10 mW test, participants were required to walk for 10 m at their normal speed (35). Time was measured for the intermediate 6 m to allow for acceleration and deceleration. Participants were encouraged to use their regular footwear and, if required, use their walking aid (36). Two trials were conducted and the average was recorded in seconds (s). Normal gait speed was categorized according to the Asian Working Group of Sarcopenia (AWGS) (37). The 10 mW test is a valid and reliable tool to assess gait speed among older adults with ICC value of 0.93 (38).

#### 2.3.7. Perceived environmental barriers assessment

In this study, CHIEF was used to document the perceived environmental barriers among older adults. CHIEF assesses the integration of environmental features and impact on outdoor participation. CHIEF is a self-administered questionnaire comprising 25 items assessing environmental factors with five subscales, which are attitude and support (five items), service and assistance (seven items), physical and structural (six items), work (three items), and policy (four items). The frequency with which a barrier is encountered is determined for each item on a subscale of 0–4 (0 = never, 1 = less than monthly, 2 = monthly, 3 = weekly, 4 = daily). If a barrier did not apply to the participant, the barrier was omitted from and did not contribute toward the calculation of the mean product score. Additionally, the magnitude of each reported barrier is quantified on a scale of 0–2, with 0 indicating no problem because the barrier was never encountered, 1 suggesting a minor issue and 2 indicating a major issue. The product of the frequency and magnitude scores (0–8) indicates the total influence of the barriers. The mean product scores of all 25 items were then used to obtain the overall perceived environmental barrier. The mean product score of the 5 subscales was further compared to determine the type of environmental barriers. From this, we obtained the top five items reported to be the greatest environmental barriers and the lowest five items reported to be the least problematic barrier faced by older adults with hearing loss. The CHIEF demonstrated strong test–retest reliability (ICC = 0.62) and internal consistency reliability (Cronbach = 0.93) in Whiteneck et al. (39), as well as indications of content, construct, and discriminant validity. We used the English and Malay versions of the CHIEF questionnaire. The Malay questionnaire was back-to-back translated and had a Cronbach alpha of 0.89.

#### 2.3.8. Outdoor mobility pattern

Participants were asked how often they went to such places as: health care institutions, places of worship, supermarkets, restaurants, and banks. Participants’ outdoor mobility was then categorized into two groups. Those who reported going outside their homes 4–7 times per week were grouped as ‘going outdoors frequently,’ while those who reported going outside less than 4 times per week were grouped as ‘going outdoors less.’ Going out at least four times a week has been linked to staying independent in activities of daily living among older adults (15).

#### 2.3.9. Transportation patterns

For each visit to the places stated in ‘Outdoor mobility pattern’ above, the participants were asked about their mode of transportation: (a) private vehicle, as driver, (b) private vehicle, as passenger, (c) public transportation, (d) e-hailing, and (e) walking. Participants were then categorized into three transportation types of groups (flexible, only use private vehicle and restricted). Those who are flexible in their choice of transportation (using public transport and/or private vehicles) were referred to as ‘flexibles,’ while those who primarily use only private vehicles were categorized as ‘only use private vehicle’ and older adults who relied fully on others or have to walk to the places were named the ‘restricted’ (40).

#### 2.3.10. Frequency of health care visits

The participants were asked about their frequency of going to hospitals, health clinics (publicly funded), and private clinics separately, ranging from 1 to 5, with 1 = every day, 2 = every week, 3 = every month, 4 = more than a month between visit and 5 = never. In this study, ‘frequent visits’ to health care were defined as going to one or multiple health centers at least once a month. For example, a participant who reported going to both hospitals and health clinics on a ‘more than once a month between visit’ basis was considered as having ‘frequent visits’ to health care institutions.

#### 2.3.11. Geriatric depression scale

Geriatric depression scale is a widely used scale to assess depression among older adults. It is a self-rating scale developed to screen for depression. Teh and Hasanah (41) omitted Item 9 in GDS-15 to develop the Malay GDS-14 scale (M-GDS-14), which has shown good psychometric uses, with Cronbach’s α coefficient of 0.84 and test–retest reliability of 0.85.

### 2.4. Data analysis

The data collected was analyzed using the Statistical Package for the Social Sciences (SPSS) version 26 application software, with *p* < 0.05 selected as the significance level. ANOVA test was performed for continuous variables to examine the differences among the three transportation groups. Chi-square test was performed for categorical variables to examine the differences between the transportation group. For categorical variables that do not meet the chi-square test assumptions, the Fisher exact test was performed. To analyze the factors associated with transportation patterns, adjusted binary logistic regression was performed.

## 3. Results

### 3.1. Characteristics of participants

**Figure 2:**
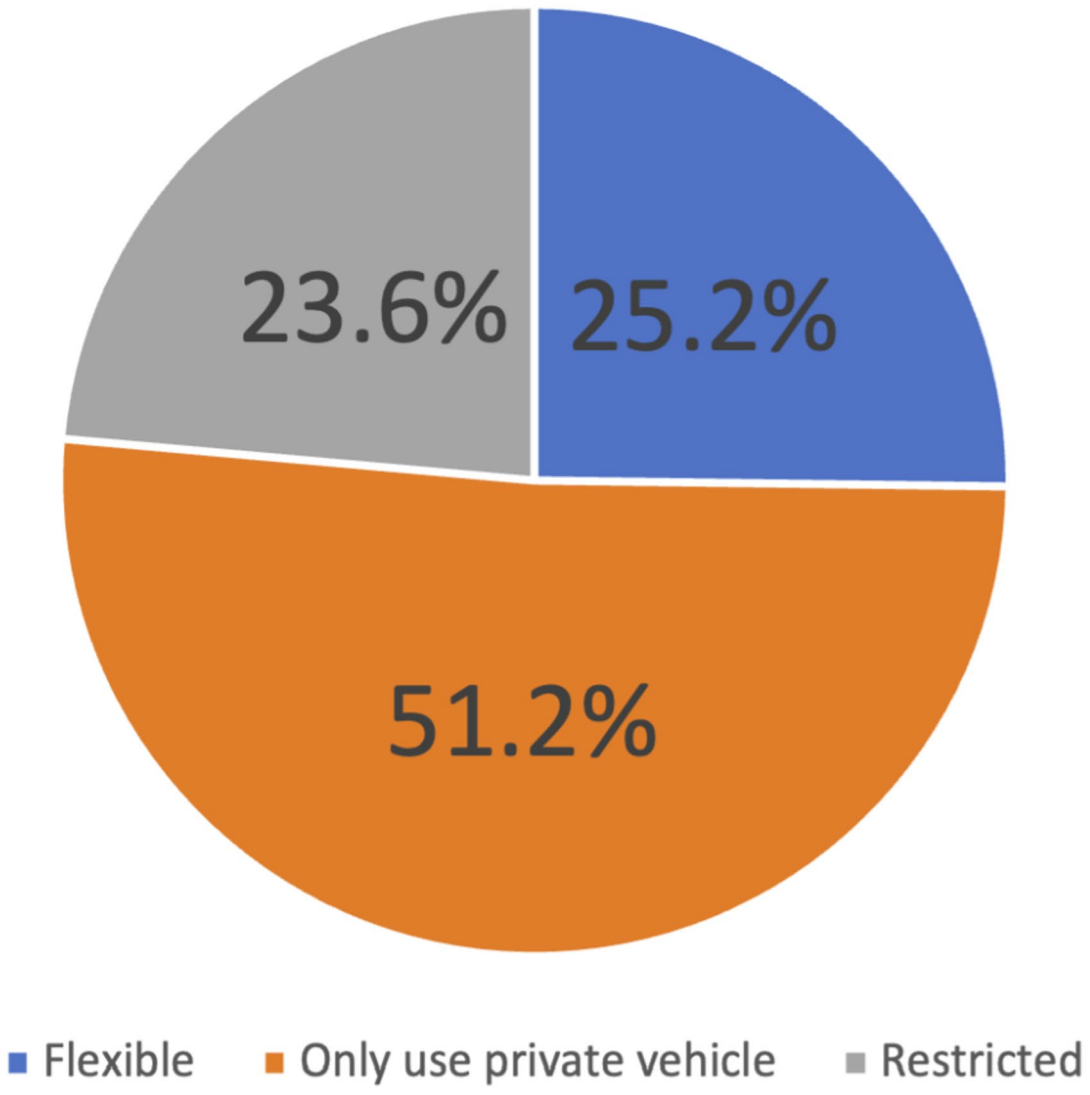
Participants’ transportation patterns.

**Figure 3:**
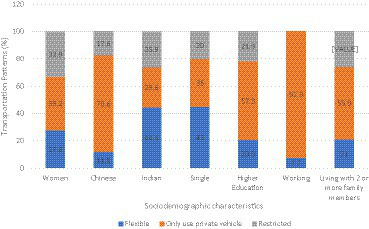
Transportation patterns according to sociodemographic characteristics.

Ten (8%) participants were excluded because of incomplete assessment, primarily the audiometry tests. A total of 119 participants were included in the study analysis. Figure 2 shows the overall transportation patterns among the 119 participants. The participants’ mean age (standard deviation) was 67.51 (5.54), with the majority (68.1%) in the 60–69 years age group. Most of the respondents (66.4%) were women. In terms of ethnicity, the majority (48.7%) were Malay. Most of the participants (66.4%) were married, had more than 6 years of formal education (80.7%), were not working or retired (89.1%), and lived with their family members (88.2%). About 56% of the participants reported having multi-morbidities such as hypertension, diabetes, and dyslipidemia. The transportation pattern groups are depicted in Figure 2. Figure 3 shows transportation patterns according to selected sociodemographic characteristics. As presented in Table 1, transportation patterns differ significantly with age, gender, ethnic groups, marital status, education level, employment status, and living status. The participants in the ‘only use private vehicle’ group were significantly younger (mean ± SD: 66.39 ± 4.54), compared to the participants in the ‘flexibles’ group who were older (mean ± SD: 69.63 ± 6.11 years). Regarding gender, significant difference was noted, as the majority (92.9%) of participants in the ‘restricted’ group were women. Regarding ethnicity, ethnic Chinese participants only use private vehicles. This pattern is reversed for ethnic Indian participants. In terms of marital status and education level, significant differences were observed in both the ‘only use private vehicle’ and ‘restricted’ groups, where participants who were single and had more education were more likely to use only private vehicles for their mode of transportation. We found that participants living with two or more family members were likely to use only private vehicles (Table 2).

**Table 1:** Sociodemographic characteristics based on transportation patterns.

Characteristics	Overall (*n* = 119)	Flexible (*n* = 30)	Only use private vehicle (*n* = 61)	Restricted (*n* = 28)	*p*-value
Age, years, mean (SD)	67.51 (5.54)	69.63 (6.11)	66.39 (4.54)	67.68 (6.34)	**0.030**
Gender, *n* (%)					**<0.001**
Men	40 (33.6)	8 (26.7)	30 (49.2)	2 (7.1)	
Women	79 (66.4)	22 (73.3)	31 (50.8)	26 (92.9)	
Ethnic group, *n* (%)					**0.017**
Malay	58 (48.7)	14 (46.7)	29 (47.5)	15 (53.6)	
Chinese	34 (28.6)	4 (13.3)	24 (39.3)	6 (21.4)	
Indian	27 (22.7)	12 (40)	8 (13.1)	7 (25.0)	
Marital status, *n* (%)					**0.002**
Single	40 (33.6)	18 (60)	14 (23)	8 (28.6)	
Married	79 (66.4)	12 (40)	47 (77)	20 (71.4)	
Income classification, *n* (%)					0.220
B40	95 (81.9)	27 (90)	44 (75.9)	24 (85.7)	
Not B40	21 (18.1)	3 (10)	14 (24.1)	4 (14.3)	
Education level, *n* (%)					**0.019**
Lower education	23 (19.3)	10 (33.3)	6 (9.8)	7 (25)	
Higher education	96 (80.7)	20 (66.7)	55 (90.2)	21 (75)	
Employment status, *n* (%)					**0.005**
Not working	106 (89.1)	29 (96.7)	49 (80.3)	28 (100)	
Working	13 (10.9)	1 (3.3)	12 (19.7)	0 (0)	
Living status, *n* (%)					**0.016**
Living alone	14 (11.8)	8 (26.7)	5 (8.2)	1 (3.6)	
Living with 2 or more family members	105 (88.2)	22 (73.3)	56 (91.8)	27 (96.4)	
Exercise, *n* (%)					0.494
Yes	78 (65.5)	17 (56.7)	42 (68.9)	19 (67.9)	
No	41 (34.5)	13 (43.3)	19 (31.1)	9 (32.1)	

Depending on the scale of measurement, *X*^2^ tests, or ANOVAs were calculated. Bold value indicate significance at *p* <0.05.

**Table 2:** Transportation patterns according to health characteristics and outdoor mobility.

Factors	Overall (*n* = 119)	Flexible (*n* = 30)	Only use private vehicle (*n* = 61)	Restricted (*n* = 28)	*p*-value
Biophysical factors					
Number of comorbidities					**0.041**
No comorbidities	25 (21)	2 (6.7)	12 (19.7)	11 (39.3)	
1–3 comorbidities	78 (65.5)	24 (80)	39 (63.9)	15 (53.6)	
4 or more comorbidities	16 (13.4)	4 (13.3)	10 (16.4)	2 (7.1)	
Anthropometric					
BMI, kg/m^2^, *n* (%)					0.978
Underweight	27 (22.7)	8 (26.7)	13 (21.3)	6 (21.4)	
Normal	44 (37)	11 (36.7)	23 (37.7)	10 (35.7)	
Overweight	48 (40.3)	11 (36.7)	25 (41)	12 (42.9)	
Malnutrition assessment, score, *n* (%)					0.409
Normal nutritional status	65 (54.6)	20 (66.7)	32 (52.5)	13 (46.4)	
At risk of malnutrition	49 (41.2)	8 (26.7)	27 (44.3)	14 (50)	
Malnourished	5 (4.2)	2 (6.7)	2 (3.3)	1 (3.6)	
Physical performance/status					
SARC-F, frail, *n* (%)	75 (63)	18 (60)	34 (55.7)	23 (82.1)	0.052
Not frail	44 (37)	12 (40)	27 (44.3)	5 (17.9)	
IADL, independent, *n* (%)	101 (84.9)	25 (83.3)	58 (95.1)	18 (64.3)	**0.001**
dependent	18 (15.1)	5 (16.7)	3 (4.9)	10 (35.7)	
Handgrip strength, normal, *n* (%)	61 (51.3)	15 (50)	28 (45.9)	18 (64.3)	0.270
Timed up and go, at risk of fall, *n* (%)	75 (64.7)	20 (66.7)	40 (65.6)	16 (57.1)	0.695
Gait speed, normal, *n* (%)	71 (59.7)	16 (53.3)	41 (67.2)	14 (50)	0.220
Outdoor mobility, frequent, *n* (%)	62 (52.1)	11 (36.7)	39 (63.9)	12 (42.9)	**0.027**
Healthcare Visit, frequent, *n* (%)	58 (56.3)	17 (60.7)	30 (57.7)	11 (42.3)	0.332
Vision					
Visual acuity, VI, n (%)	37 (31.1)	8 (26.7)	20 (32.8)	9 (32.1)	0.831
Contrast sensitivity, log CS, mean (SD)	1.47 (0.18)	1.45 (0.23)	1.50 (0.14)	1.45 (0.20)	0.335
Hearing					
Four frequency average, HL, *n* (%)	19 (16)	5 (16.7)	8 (13.1)	6 (21.4)	0.576
Five high frequency Average, HL, *n* (%)	43 (36.1)	9 (30)	25 (41)	9 (32.1)	0.521
Psychological factors					
GDS, depression, *n* (%)	25 (21)	4 (13.3)	12 (19.7)	9 (32.1)	0.200
IDEA, mean (SD)	13.94 (1.62)	13.27 (1.89)	14.21 (1.57)	14.07 (1.18)	**0.027**
Fear of fall, *n* (%)					**0.035**
Yes	52 (43.7)	15 (50)	20 (32.8)	17 (60.7)	
No	67 (56.3)	15 (50)	41 (67.2)	11 (39.3)	

Depending on the scale of measurement, *X*^2^ tests, or ANOVAs were calculated. VI, vision impairment; HL, hearing loss. Bold value indicate significance at *p* <0.05.

### 3.2. Health characteristics and outdoor mobility stratified by transportation patterns

Significant differences were found among the transportation groups in terms of age, number of comorbidities, independence in IADL, outdoor mobility pattern, cognitive function, and fear of falling. The ‘only use private vehicle’ group was significantly different from the ‘restricted’ group in terms of IADL independence and fear of falling. Participants who were not fully dependent and have fear of falling were more likely to be in the ‘restricted’ group. In terms of number of comorbidities, a significant difference was noted in the ‘restricted’ group, where a majority (53.6%) of the participants had one to three comorbidities. The *Z*-test further showed that the ‘flexible’ and the ‘only use private vehicle’ groups differed significantly in terms of age and cognitive function, where the participants in the ‘flexible’ were older and had lower cognitive function score.

### 3.3. Environmental barriers score stratified by transportation patterns

Table 3 shows the transportation patterns according to environmental factors and its sub-domains. Physical barriers appeared to be the greatest barrier reported by the participants with mean (standard deviation) of 0.86 (1.12). However, no significant difference was reported across transportation patterns in all sub-domains.

**Table 3:** Transportation Patterns according to environmental barriers.

Environmental factors	Overall (*n* = 119)	Flexible (*n* = 30)	Only use private vehicle (*n* = 61)	Restricted (*n* = 28)	*p*-value
CHIEF total score	0.52 (0.58)	0.62 (0.64)	0.46 (0.59)	0.57 (0.48)	0.401
Physical barriers	0.86 (1.12)	1.05 (1.04)	0.64 (0.97)	1.12 (1.43)	0.097
Attitude barriers	0.29 (0.50)	0.29 (0.45)	0.29 (0.55)	0.29 (0.43)	0.999
Service barriers	0.47 (0.71)	0.61 (0.93)	0.40 (0.64)	0.47 (0.56)	0.434
Policies barriers	0.47 (0.88)	0.55 (0.96)	0.49 (0.92)	0.35 (0.68)	0.671
Work barriers	0.51 (0.80)	–	0.40 (0.58)	0.73 (1.16)	0.465

ANOVA analysis.

### 3.4. Association between transportation patterns and health characteristics and outdoor mobility

The binary logistic regression model (Table 4) indicates that gender, number of comorbidities, and IADL are significant predictors of independence in transportation [Chi-Square = 37.180, df = 11 and *p* = 0.001 (<0.05)]. The other eight predictors, namely, age, ethnic group, marital status, education level, living group, cognitive (IDEA), fear of falling and outdoor mobility are not significant. Gender, number of comorbidities and IADL are significant at the 5% level [gender Wald = 7.380, *p* = 0.007 (<0.05); number of comorbidities Wald = 7.278, *p* = 0.007 (<0.05); IADL Wald = 9.913, *p* = 0.002 (<0.05)]. The odds ratio (OR) for significant predictors are: gender [0.090 (95% CI: 0.016–0.512)]; number of comorbidities [4.003 (95% CI: 1.461–10.967)]; and IADL [11.085 (95% CI: 2.480–49.5540)]. The model correctly predicted 46.4% of cases in ‘restricted’ group and 95.6% of cases in ‘other transport’ group, giving and overall percentage correct prediction rate of 84%.

**Table 4:** Factors associated with transportation patterns.

Variable	β	SE β	Wald’s χ^2^	*p*	Odds ratios (*e*^β^)	95% CI
Age	0.020	0.056	0.125	0.724	1.020	0.913–1.139
Gender	−2.406	0.886	7.380	**0.007**	0.090	0.016–0.512
Ethnic group	−0.528	0.432	1.490	0.222	0.590	0.253–1.376
Marital status	−1.071	0.675	2.515	0.113	0.343	0.091–1.288
Education level	0.003	0.798	0.000	0.997	1.003	0.210–4.790
Living group	−0.164	0.428	0.147	0.701	0.848	0.367–1.964
Cognitive (IDEA)	−0.016	0.203	0.006	0.937	0.984	0.662–1.464
Number of comorbidities	1.387	0.514	7.278	**0.007**	4.003	1.461–10.967
IADL	2.406	0.764	9.913	**0.002**	11.085	2.480–49.554
Fear of fall	−0.721	0.572	1.592	0.207	0.486	0.159–1.491
Outdoor mobility	0.160	0.627	0.065	0.779	1.173	0.343–4.009

Binary logistic regression analysis. Refers to significance at 1% level. SE, standard error; CI, confidence interval. Bold value indicate significance at *p* <0.05.

## 4. Discussion

Our study examined the biopsychosocial factors that affected the transportation patterns of older adults in an urban center in Malaysia, a low to middle income country (LMIC). Referring to the biopsychosocial framework (24), we examined the impact of biophysical (physical, vision, and hearing ability), psychological (symptoms of depression and lack of cognitive function), and social (income status, education level, marital status, living status) factors on older adults’ transportation patterns. Being female, having a higher number of comorbidities, and lower independence in IADL are significantly associated with the older adults’ dependence on transportation.

A majority of the older adults in our study were in the ‘only use private vehicles’ group. The adults in this group were younger, compared to those in the ‘restricted’ and ‘flexible’ groups. This finding is supported by a recent review which found that older Malaysians are moving from using public transport to driving private vehicles (42). Moreover, older Malaysian adults are more likely to be driving, compared to older adults from other Asian countries, namely Singapore, Thailand, Japan, and Korea (43–45). The reason for this move is not clear but may be related to the ease of using private vehicles instead of public transportation. It is also noteworthy that the adults in the ‘only use private vehicle’ group went out more frequently than the adults in the ‘restricted’ group. This is to be expected as the adults in the ‘only use private vehicles’ group were able to drive.

The majority of the older adults in the ‘restricted’ group are females, Malay, married, not working and living with two or more family members. However, when the ‘flexible’ and the ‘restricted’ groups are compared, it is noted that the adults in the latter group go out more. The adults in the ‘restricted’ group probably have good social support from family members (based on their living status information) for transportation. Good social support, whether from family or the neighborhood/community, has been recognized as an enabler for frequent outdoor mobility, even when infrastructure capital, that is, car ownership, driving license, and good roads are not available (46–48).

After adjusting for covariates, our study showed significant association among transportation patterns and gender, number of comorbidities, and IADL. We found that women are more reliant on others for transportation. This finding is consistent with a previous study where older women were more likely to have transportation problems and rely on public transportation (18). In LMICs, the majority of public transport users are women, many of whom do not own cars or stopped driving prematurely (49, 50). Gender differences in travel patterns and the use of public transportation have been reported previously (51, 52). There may also be a socio-cultural bias against women traveling alone but there is limited evidence supporting this proposition in Malaysia.

In terms of ethnicity, older Indian adults are more open to using various modes of transportations, compared to the majority of Malays and Chinese who only use private vehicles in this study. This may be related to the lower socioeconomic status of Indian participants. For example, our results showed that a higher percentage of the older adults in the ‘flexible’ group were of lower socioeconomic status. However, the relationship of being of lower socioeconomic status and being more flexible in the use of transportation is not clear. More information is required.

Older adults with a higher number of comorbidities (60.7%) and lower scores for IADL (64.3%) were more restricted in their use of transportation. Dependency among older adults is often the result of health status, activity and participation, personal and environmental factors (53). A previous local study has shown the relationship between dependency in IADL and low socioeconomic status (54). Older adults in poor health and functional decline may be more dependent on others for outdoor mobility and transportation (55). There is a possible two-way association among health, functional statuses and transportation reliance. For example, older adults who are less able to travel outside their homes may be in poorer health and wellbeing (56), while older adults who are traveling outside more and who use public transportation gained multiple nutritional and health benefits (57).

In this study, we found no association between transportation patterns and physical performance, vision and hearing status. Overall, the mean TUG scores and 10 mW test performance of the participants were within the norms (58). Living in an urban center may have helped. A study in Japan indicated that individuals living in urban centers have better lower limb strength compared to those living in rural areas. This may be influenced by higher physical activity engagement among urban individuals compared to rural individuals as reported in the study (59). It could also be possible that there is adequate community accessibility in this area and hence increased mobility as demonstrated in a local study among the older adults (60).

Visual impairment was not associated with transportation patterns among our study participants. Similarly, in a previous report, visual impairment did not have impact on the functional mobility of older adults (61). In another local study, participants with reduced visual acuity and contrast sensitivity continued driving actively (64). Likewise, no significant association was found between transportation patterns and hearing impairment among the older adults in our study. Perhaps the majority of the participants in this study have relatively good hearing ability with only 36% of participants having hearing loss at five high frequency average. However, it should be noted that previous studies have found a clear association between reduced hearing abilities and mobility-related difficulties, which may even lead to falls (62, 63). To date, Malaysia does not have an age limit or age-related test for the issuance of drivers’ licenses for older drivers. Perhaps, age-related tests need to be implemented to ensure only older adults that are still fit to drive can get their licenses renewed.

These conflicting results could be due to the different methodologies used, study locations and types of audiovisual tests performed. Our study results did not find any disparity between the frequency of health care visits and outdoor mobility and transportation patterns, which suggests that, despite the older adults having reduced outdoor mobility and restricted transportation, they were still meeting their need for health care visits. In a recent review regarding older adults’ health care access issues in Southeast Asia, high transportation cost and low social/family support were two factors highlighted as barriers to health care (23). It is also noteworthy that older adults with spouses are more likely to access healthcare, compared to those who are alone, as shown in a local study examining factors associated with healthcare access (20). This highlights the crucial informal support from family in meeting older adults’ healthcare needs. In Malaysia, community transport services for people requiring ongoing access to healthcare are not available nationwide. This need to be addressed to ensure informal support is not the only viable alternative to meet their needs (46).

Although no significant difference found across transportation groups for environmental factors, our study identified physical barriers as the most difficult environmental/social factors encountered by older adults across all transportation group. This finding was also confirmed in previous studies (47, 65), which suggests that poor facilities and infrastructure in the older adults’ environment influence their perception of their environment. However, this finding needs to be considered with caution as we used self-perceived environmental barriers rather than objectively measured environmental barriers. Another limitation was that we did not record the participants’ unmet healthcare and outdoor mobility needs. This information would have improved understanding of the role of transportation patterns for health care visits and outdoor mobility. Also, being a cross-sectional survey data, it limits the causal effect inferences of aging and its impact on transportation.

## 5. Conclusion and implications

Our findings indicate that older Malaysian adults residing in the Klang Valley rely primarily on private vehicles for their transportation needs. Further information and research is required to meet the transportation needs of older adults, in particular, older women, those with comorbidities and those with decreased independence. Transportation policies that consider the needs of older adults and improved public transportation services that meet the needs of older adults may help these older adults maintain their independence.

## Data availability statement

The raw data supporting the conclusions of this article will be made available by the authors, without undue reservation.

## Ethics statement

The studies involving human participants were reviewed and approved by Research Ethics Committee of Universiti Kebangsaan Malaysia (UKM PPI/111/8/JEP-2021-742). The patients/participants provided their written informed consent to participate in this study.

## Author contributions

NM, DS, WI, and MA researched and analyzed the background literature. SS, CM, and TH critically provided substantial scholarly guidance on the manuscript draft and interpretation for intellectual content. AM and AA provided the statistical guidance. All authors approved the final version of the manuscripts, ensure the accuracy and integrity of the work.

## Funding

The authors acknowledge financial support from the Ministry of Higher Education Malaysia under a Consortium on Mobility and Transportation in an Ageing Society (CoMTAS) [JPT(BKPI)1000/016/018/25(55)] grant (KKP/2020/UPM-UKM/8/2).

## Conflict of interest

The authors declare that the research was conducted in the absence of any commercial or financial relationships that could be construed as a potential conflict of interest.

## Publisher’s note

All claims expressed in this article are solely those of the authors and do not necessarily represent those of their affiliated organizations, or those of the publisher, the editors and the reviewers. Any product that may be evaluated in this article, or claim that may be made by its manufacturer, is not guaranteed or endorsed by the publisher.
